# Rates, perceptions and predictors of depression, anxiety and Post Traumatic Stress Disorder (PTSD)-like symptoms about Covid-19 in adolescents

**DOI:** 10.1371/journal.pone.0266818

**Published:** 2022-04-27

**Authors:** Tracy M. Stewart, Debi Fry, Lesley McAra, Sarah Hamilton, Albert King, Margaret Laurie, Gillean McCluskey

**Affiliations:** 1 Moray House School of Education and Sport, University of Edinburgh, Edinburgh, Scotland; 2 Edinburgh Law School, University of Edinburgh, Edinburgh, Scotland; 3 UNICEF, London, United Kingdom; 4 Scottish Government, Edinburgh, Scotland; Fondazione Istituto Neurologico Nazionale C Mondino Istituto di Ricovero e Cura a Carattere Scientifico, ITALY

## Abstract

Increasing evidence has shown that the Covid-19 outbreak, and subsequent school closures and exam cancellations that followed, has impacted adolescent mental health. The current cross-sectional study examined rates of depression, anxiety and Post Traumatic Stress Disorder (PTSD)-like symptoms about Covid-19 in adolescents and whether current or past mental health support, additional support in school, keyworker status, poorer household relationships since the outbreak of Covid-19 or reduced physical activity were associated with elevated levels of depression, anxiety or PTSD-like symptoms. Lastly, it examined perceived changes in mental health due to the Covid-19 pandemic, school closures and the cancellation of exams. A total of 899 adolescents (14–18 years) took part in the ’in isolation instead of in school’ (INISS) project. Findings indicated that older adolescents, females, those who currently or previously received mental health support or additional support in school and adolescents who reported poorer relationships at home since Covid-19 were more likely to meet clinical threshold levels for their mental health. Adolescents highlighted worsening of their mental health due to Covid-19 and school closures with mixed positive and negative impact of exam cancellations. Adolescents experiencing clinical threshold levels of depression and anxiety uniquely reported worsening of their mental health since the Covid-19 pandemic, school closures and exam cancellations. Understanding the rates, perceptions and factors associated with increases in depression, anxiety and PTSD-like symptoms in adolescents during the Covid-19 pandemic will inform national policy in supporting adolescent mental health and recovery from the Covid-19 pandemic.

## Introduction

Research evidence on the impact of the Covid-19 pandemic on global mental health has been accelerating over the past 18 months, particularly in the adult literature. However, despite calls that children and adolescents are being left behind in Covid-19 mental health research [1] and the United Nations advocacy for “a rapid accumulation of data on the scale and nature of impacts among children” [2], mental health research with children and adolescents has been slower to emerge. Understanding the mental health consequences, specifically for children and adolescents, in the context of Covid-19 is important, as previous research has suggested that pandemics, emergencies and disasters can disproportionately affect children and adolescents. For example, in addition to experiencing trauma related to the event, adolescents often lose essential resilience supporting factors such as the social infrastructure that would normally be in place to safeguard and support them [3].

Cross-sectional studies during the early stages of the pandemic provided early evidence that the Covid-19 pandemic has negatively impacted the mental health of children and adolescents. For example, studies from China reported that 22.6% of primary-aged school children had symptoms of depression and 18.9% symptoms of anxiety [4] and 43.7% symptoms of depression and 37.4% symptoms of anxiety in adolescents [5], which are greater than pre-pandemic rates [6, 7]. In England, Mansfield et al. [8] found that 14% of adolescents (aged 12–21 years) met clinical threshold cut-off levels for depression and 10% anxiety. While using different measures, this is comparably greater than pre-pandemic depression data with children and adolescents (aged 5–19 years) in England where 2.1% met diagnostic criteria for depression and 7.2% anxiety [9]. In Malaysia, Zainudeen et al. [10] reported that 25.4% of children and adolescents (aged 5–18 years) had elevated post-traumatic stress disorder (PTSD)-like symptoms of avoidance and intrusive thoughts. Similarly, in Italy, Davico et al. [11] reported that rates of avoidance and intrusive thoughts were 30.9% for young people aged 8–18 years old. While not directly comparable (as the avoidance and intrusive thoughts measure is not a diagnostic measure), lifetime rates of PTSD have been shown to be between 3%-8%, with a study in England and Wales reporting rates of PTSD at 7.8% in 18 year old adolescents [12].

In studies with pre-and-during Covid-19 pandemic data, a similar pattern is emerging of declining mental health in children and adolescents during the Covid-19 pandemic. Research from Australia reported increases in depression and anxiety symptoms in 13 to 16 year-olds from pre-pandemic to two months into the pandemic (two months imposed the stay-at-home orders and online learning for schools) [13]. In an Icelandic sample of adolescents (aged 13–18 years) depression symptoms increased and wellbeing reduced during the Covid-19 pandemic, more so than would be expected based on previous data trends [14]. In the USA, Rosen et al. [15] (2021) reported that 31.7% of adolescents (aged 13–15 years) met subclinical or clinical thresholds for internalising problems and 17.4% externalising problems pre-pandemic, rising to 56.7% for internalizing problems and 56.2% for externalising problems at the beginning of the pandemic (6 months later during the stay-at-home orders). A similar trend has been reported in 12 longitudinal studies (10 x USA, 1 x the Netherlands and 1 x Peru studies) with pre-and-during pandemic data with children and adolescents (aged 9–18 years), with the authors reporting that collectively, depression symptoms increased by 28% however anxiety symptoms remained stable [16].

In the UK, data from England has shown an increase of 5.2% in probable mental health difficulties in children and young people aged 5–16 years, rising from 10.8% in 2017 to 16% in July 2020 [17]. In a further study, Wright et al. [18] reported a 44% increase of depression symptoms and 26% for post-traumatic stress symptoms in 11–12 year olds pre-Covid-19 to after the first lockdown. They also found parent reported increases in child depression and child PTSD symptoms but no changes to child anxiety symptoms. Furthermore, Bignardi et al. [19] found that depression symptoms increased substantially during lockdown (April—June 2020) in 7–12 year olds in England but no significant changes in anxiety symptoms. Widnall et al. [20] however, reported no changes in depressive symptoms and a small decrease in anxiety symptoms in adolescents aged 13–14 years in England from October 2019 to Spring 2020. While less research has been conducted in a Scottish context, one adult study that included participants from Scotland, England, Wales and Northern Ireland, reported that older adolescents (aged 18–24 years) displayed increased mental distress during Covid-19, which was greater than expected from trends observed before the pandemic [21].

There is also emerging evidence of the psychological impact of Covid-19 for particular groups of children and adolescents, such as children with special educational needs and disabilities [22] and children and young people with pre-existing mental health difficulties [23]. For example, Zijlmans et al. [23] recruited three groups of 8–18 year olds from three sources; from a psychiatric cohort (recruited from psychiatric clinics) a paediatric cohort (recruited from hospital all under treatment for chronic somatic conditions), and a general population cohort (recruited online). The authors found that the psychiatric cohort reported the worst mental health, the paediatric cohort reported the best, whereas the general population cohort had scores in between (assessed during the first lockdown in the Netherlands April/May 2020). Children and adolescents from the general population cohort also reported a worse atmosphere at home during the COVID-19 lockdown and in a qualitative aspect to the study, all three groups reported that the Covid-19 pandemic negatively impacted their lives, ranging from missing their friends to not being allowed to go to school and participate in sports.

The negative impact of the Covid-19 pandemic on adolescent mental health is not surprising given the mass quarantines, school closures, exam cancellations and social distancing mitigation measures brought about by the Covid-19 pandemic. These measures can be particularly challenging for adolescents who are within a stage of development characterised by rises in mental health difficulties, parental conflict and a developmental need associated with social connection and acceptance from peers [24–27]. While less is known about the impact that school closures and exam cancelations had on children and adolescents’ mental health and wellbeing, preliminary research has shown the negative impact of Covid-19 related restrictions. For example, one qualitative study with parent/child-adolescent dyads in Ireland, reported increased feelings of depression and anxiety, stress of home-schooling and feelings of isolation [28]. In another qualitative study, Scottish adolescents reported the immediate and complete loss of social contact with peers during lockdown and the negative impact that had on their mental health [29]. Similarly, in a study conducted by the Scottish Youth Parliament, YouthLink Scotland and Young Scot [30], the authors reported that adolescents were concerned about their own and others mental wellbeing, school closures, exams and coursework.

The Organisation for Economic Co-operation and Development (OECD) [31] highlighted that school closures may have significant implications not only for mental wellbeing of children and young people but that closures resulted in an erosion of protective factors that schooling offers, such as social contact, emotional support from teachers, a sense of belonging and access to physical exercise. Indeed, children and young people with pre-existing mental health conditions and those who received additional support in school, may be uniquely vulnerable due to these rapid and ongoing changes in routines, service and educational access. To inform practice and policy on the impact on adolescence from the outbreak of Covid-19 in Scotland, the ‘in isolation instead of in school’ (INISS) project, a multi-method, multidisciplinary (Psychology, Education, Law and Sociology) and multi-industry (partnered with the Scottish Government and UNICEF-UK) project was devised to investigate education and mental health outcomes in young people during Covid-19. The data reported in this paper pertains to one aspect of the wider INISS project, which aimed to investigate three main research questions:

**RQ1:** What are the rates of depression, anxiety and PTSD-like symptoms about Covid-19 in adolescents during the Covid-19 pandemic?
**RQ2:** Are adolescents who (i) receive current or past mental health support, (ii) receive additional support in school, (iii) are themselves (or parents / carers) keyworkers, (iv) have poorer household relationships since the outbreak of Covid-19, or those who (v) engaged in reduced physical activity since the outbreak of Covid-19 more likely to meet clinical threshold levels for their mental health?
**RQ3:** Do adolescents perceive their mental health and wellbeing to have been impacted by school closures, exam cancelations and / or the outbreak of Covid-19?

## Materials and methods

### Participants

A total of 1003 participants consented to take part in the INISS study overall. A total of 75 participants (7.4%) did not provide any data and were removed from the dataset. A further 29 participants (2.9%) were removed at the data cleaning stage due to offensive or inconsistent responses. The final sample consisted of 899 adolescents aged 14–18 years old (*M*_age_ = 15.89 years, *SD* = 0.88). Over half identified as female (51%, *n* = 462), almost a third as male (30%, *n* = 267) and a small number identified as non-binary (1%, *n* = 8) with one participant stating they were unsure of their gender (0.1%, *n* = 1). In terms of trans status, one participant (0.1%) identified as trans. The remaining 18% of participants (*n* = 160) did not disclose their gender. Ethnicity was as follows: White (70%, *n* = 625), Asian or Asian British (5%, *n* = 46), Mixed (3%, *n* = 28), African (2%, *n* = 15), Arab (1%, *n* = 9) or Caribbean or Black (1%, *n* = 5). The remaining 19% (*n* = 171) participants did not specify their ethnicity. The Scottish Index of Multiple Deprivation (SIMD) ranging from quintile 1 (obtaining the 20% most deprived data zones in Scotland) to quintile 5 (obtaining the 20% least deprived data zones in Scotland) classified the sample as follows: quintile 1 = 8% (*n* = 71), quintile 2 = 6% (*n* = 58), quintile 3 = 14% (*n* = 129), quintile 4 = 15% (*n* = 135) and quintile 5 = 29% (*n* = 263). We could not obtain SIMD scores from 27% (*n* = 243) of participants due to incomplete postcode data.

### Procedure

Ethical approval was obtained from Moray House School of Education and Sport, University of Edinburgh. A research advisory group consisting of three young people and three external research experts advised on the project. An online survey was created, hosted on GLOW, the website for the Scottish Government’s national education agency, known as Education Scotland, and was available to all school students, teachers and parents in Scotland. All secondary schools in Scotland were contacted via email with study details and access routes to GLOW. The survey was also accessed through a variety of additional media, e.g. on the INISS dedicated website, and through Scottish Youth Parliament, YouthLink and Pupil Inclusion Network websites. The survey was open to pupils, aged between 14 and 18 years old, between August and September 2020, which coincided with the return of Scottish pupils to school after the summer school break. The survey items were randomised to minimise response bias and the survey length kept as short as possible for accessibility reasons and to minimise non-response bias.

### Measures

#### Depression

Assessed using the Revised Children’s Anxiety and Depression Scale Short Version for Children (RCADS-25) depression subscale [32]. The RCADS-25 is a short self-report questionnaire for children and adolescents aged 8 to 18 years old that measures levels of depression through 10 items. Items are scored on a four-point Likert scale: 0 = never, 1 = sometimes, 2 = often, and 3 = always, resulting in a range of total scores from 0 to 30. The scale had excellent reliability (*α* = .92). A binary clinical depression threshold outcome was set using the diagnostic thresholds (t‐scores ≥ 70) [33].

#### Anxiety

Assessed using the Revised Children’s Anxiety and Depression Scale Short Version for Children (RCADS-25) anxiety subscale [32]. The RCADS-25 is a short self-report questionnaire for children and adolescents aged 8 to 18 years old that measures broad anxiety through 15 items. Items are scored on a four-point Likert scale: 0 = never, 1 = sometimes, 2 = often, and 3 = always, resulting in a range of total scores from 0 to 45. The scale had excellent reliability (*α* = .88). A binary clinical anxiety threshold outcome was set using the diagnostic thresholds (t‐scores ≥ 70) [33].

#### Avoidance and intrusion

Assessed by an edited version of the Child Revised Impact of Events Scale short version (CRIES-8) [34]. The CRIES-8 is a short self-report questionnaire for children and adolescents aged 8 years and above that measures risk for PTSD through 8 items. No wording of items were edited, however “thinking about Covid-19” was added to the pre-faced text: *“below is a list of comments made by people after stressful life events*. *[Thinking about Covid-19]*, *please click on the responses for each item showing how frequently these comments were true for you during the past seven days*. *If they did not occur during that time please click the ‘not at all’ box*.*”* This allowed us to measure the frequency of avoidance and intrusive thoughts in relation to Covid-19. Items are scored on a four-point Likert scale: 0 = not at all, 1 = rarely, 3 = sometimes, and 5 = often, resulting in a range of total scores from 0 to 40. The scale has two 4-item subscales, instruction and avoidance, with total scores on each ranging from 0 to 20. The scale had excellent reliability (*α* = .89). A binary elevated avoidance and intrusion outcome was set using a cut-off score of 17 [34].

#### Perceived changes in depression, anxiety and wellbeing

Assessed by three questions directly after completing the Short Warwick-Edinburgh Mental Wellbeing Scale (SWEMWBS) [35], repeated after the RCADS depression sub-scale and again after the RCADS anxiety subscale “*Since the Covid-19 outbreak*, *have these things been worse*, *the same*, *or better than before*?*”*, *“Did the exam cancellations make these things worse*, *the same*, *or better than before*?*”* and *“Did your school closing to most pupils make these things worse*, *the same*, *or better than before*?*”* The response options for each question were: 0 = much worse, 1 = a bit worse, 2 = the same, 3 = a bit better, 4 = much better.

#### Additional support in school

Assessed by a question devised by the authors; *Did you have any kind of regular additional support in school before the Covid-19 outbreak*?*”* The response options were: 0 = no, 1 = not sure, and 2 = Yes. A binary predictor was used for the regression analyses (0 = no, 1 = yes) with ‘not sure’ responses treated as missing.

#### Mental health support

Assessed by a single question edited from the Oxwell study [36]; *“Have you ever received any mental health support*? *For example*, *from an adult at school / CAMHS / private psychologist / counsellor / charity service*.*”* The response options were: 0 = no, 1 = yes, I am currently receiving support, 2 = yes, I have had support in the past” and 3 = not sure. A binary predictor was used for the regression analyses (0 = no, 1 = yes) with ‘not sure’ responses treated as missing.

#### Keyworker

Assessed by a single question devised by the authors; *“Are you*, *or anyone you live with*, *a keyworker*? *e*.*g*. *nurse*, *carer*.*”* The response options were: 0 = no, 1 = yes, 2 = don’t know. A binary predictor was used for the regression analyses (0 = no, 1 = yes) with ‘don’t know’ responses treated as missing.

#### Household relationships

Assessed by a single question edited from the Oxwell study [36]; *“Since Covid-19*, *have you got along less well*, *the same*, *or better with other people in your household*?*”* The response options were: 0 = much less, 1 = a bit less, 2 = the same, 3 = a bit better, 4 = much better. Poor home relationships (0 = no, 1 = yes) were determined by a binary predictor “much less” referenced against those who got along with other people in their household a bit less, the same, a bit or much better since the Covid-19 pandemic.

#### Physical activity

Assessed by a single question devised by the authors; “*Since the start of Covid-19*, *have you been physically active less*, *the same*, *or more than before*?*”* The response options were: 0 = much less, 1 = slightly less, 2 = the same, 3 = slightly more, 4 = much more. Much reduced physical activity since the Covid-19 pandemic (0 = no, 1 = yes) was determined by a binary predictor “much less” referenced against those who were slightly less, the same, slightly or much more physically active since the Covid-19 pandemic.

### Analytical strategy

Clinical threshold cut off data is reported for the sample for participants who provided data on measures of depression (*n* = 807), anxiety (*n* = 805), and PTSD-like symptoms about Covid-19 (*n* = 804). Rates are reported by gender where participants have provided data on gender and mental health outcomes, (*n* = 737). Logistic regression analysis conducted in SPSS 25 was used to examine predictors of clinical threshold levels of depression and anxiety and elevated levels of PTSD-like symptoms about Covid-19. Specifically, depression, anxiety and PTSD-like symptoms about Covid-19 were regressed on demographic control measures, including female gender (male gender as reference group), age and SIMD, alongside potential risk factors, including current or past mental health support, additional support in school, keyworker status, poor home relationship since the Covid-19 outbreak, and reduced physical activity since the outbreak of Covid-19. We report odds ratios (OR), 95% confidence intervals and significance levels to provide an estimate of the increased likelihood of adolescents meeting clinical threshold for depression, anxiety and PTSD-like symptoms about Covid-19. Missing data was identified (4% - 27%) on all study measures (including drop out data). No individual values were missing from any mental health outcome measure. The data contained a total of 72% (*n* = 646) complete cases (reducing to 62%, *n* = 554 when treating ‘don’t know’ and ‘not sure’ responses as missing data). Missing data analysis showed that data were missing completely at random (Little’s MCAR; *χ^2^* = 43.007, df = 38, *p* = .26). Participants with missing data for one or more of the variables were not included in the logistic regressions and male gender was referenced against female gender, with 542 participants (males = 187, female = 355, *M*_age_ = 15.94 years, *SD* = 0.87) included in the logistic regression models.

Perceptions of change in mental health is reported for the sample who provided data on change questions; perceived change in depression symptoms (*n* = 807), perceived change in anxiety symptoms (*n* = 805), and perceived change in wellbeing (*n* = 794). To examine statistical differences in perceived changes in mental health by gender (male/female) and clinical status, chi-square analyses were conducted. Rates are reported by gender where participants have provided data on gender and perceived mental health outcomes (*n* = 729). Rates are reported by clinical status where participants have provided data on a mental health measure and perceived mental health outcomes; depression (*n* = 807), anxiety (*n* = 805) and wellbeing (*n* = 807).

## Results

### Mental health rates

The percentage of adolescents meeting clinical threshold level for depression was 9% (13% when including borderline threshold cases), 7% met clinical threshold for anxiety (13% when including borderline threshold cases), and 29% of young people had elevated PTSD-like symptoms about Covid-19. Of the 462 females who provided data on gender and rates of depression, anxiety and PTSD-like symptoms about Covid-19, 11% (*n* = 51) met clinical threshold levels for depression, 9% (*n* = 40) anxiety and 34% (*n* = 156) had elevated PTSD-like symptoms about Covid-19. Of the 267 males who provided data on gender and rates of depression, anxiety and PTSD-like symptoms about Covid-19, 5% (*n* = 14) met clinical threshold levels for depression, 5% (*n* = 14) anxiety and 18% (*n* = 49) had elevated PTSD-like symptoms about Covid-19. Of the eight participants who identified as non-binary, 13% (*n* = 1) met clinical threshold levels for depression, 13% (*n* = 1) anxiety and 40% (*n* = 3) had elevated PTSD-like symptoms about Covid-19. Given low group membership and the potential for identification, we do not report clinical levels for participants who were unsure of their gender (*n* = 1) or those who identified as trans (*n* = 1).

### Predictors of depression, anxiety, and avoidance and intrusion

Clinical threshold levels of depression, anxiety and PTSD-like symptoms about Covid-19 were regressed on demographic control measures, including female gender (male gender as reference group), age and SIMD, alongside potential risk factors, including current or past mental health support (no = 0, yes = 1), additional support in school (no = 0, yes = 1), keyworker status (no = 0, yes = 1), poor home relationships since the outbreak of Covid-19 (no = 0, yes = 1), and less physical activity since the outbreak of Covid-19 (no = 0, yes = 1) (see Table 1).

**Table 1:** Logistic regression of depression, anxiety and avoidance and intrusion with adjusted odds ratio.

Outcomes (*n* = 542)		Independent variable	Adjusted odds ratio	95% CI	*p*
**Depression**	Step 1	Gender female (*n* = 355)	2.00	0.98, 4.06	.057
		Age (*range* = 14–18)	2.55	1.74, 3.75	**< .001**
		SIMD (*range* = quintile 1–5)	0.92	0.74, 1.14	.430
	Step 2	Gender female (*n* = 355)	1.48	0.68, 3.24	.392
		Age (*range* = 14–18)	2.97	1.90, 4.59	**< .001**
		SIMD (*range* = quintile 1–5)	0.92	0.72, 1.16	.462
		Current/past mental health support (*n* = 121)	3.76	1.83, 7.72	**< .001**
		Additional support for learning in school (*n* = 85)	3.22	1.52, 6.84	**.002**
		Keyworkers (*n* = 206)	0.97	0.49, 1.99	.971
		Poor relationships at home since Covid-19 (*n* = 35)	8.59	3.08, 23.97	**< .001**
		Much reduced physical activity since Covid-19 (*n* = 107)	1.64	0.76, 3.50	.208
**Anxiety**	Step 1	Gender female (*n* = 355)	1.74	0.77, 3.96	.185
		Age (*range* = 14–18)	2.00	1.29, 3.10	**.002**
		SIMD (*range* = quintile 1–5)	0.79	0.62, 1.01	**.059**
	Step 2	Gender female (*n* = 355)	1.30	0.55, 3.09	.551
		Age (*range* = 14–18)	2.03	1.30, 3.18	**.002**
		SIMD (*range* = quintile 1–5)	0.80	0.63, 1.03	.082
		Current/past mental health support (*n* = 121)	3.50	1.58, 7.80	**.002**
		Additional support for learning in school (*n* = 85)	1.62	0.70, 3.79	.263
		Keyworkers (*n* = 206)	0.98	0.46, 2.01	.956
		Poor relationships at home since Covid-19 (*n* = 35)	3.48	1.14, 10.63	**.029**
		Much reduced physical activity since Covid-19 (*n* = 107)	1.02	0.42, 2.45	.973
**PTSD-like symptoms**	Step 1	Gender female (*n* = 355)	2.63	1.67, 4.14	**< .001**
		Age (*range* = 14–18)	1.04	0.82. 1.30	.770
		SIMD (*range* = quintile 1–5)	0.96	0.83. 1.12	.620
	Step 2	Gender female (*n* = 355)	2.49	01.56, 3.97	**< .001**
		Age (*range* = 14–18)	1.05	0.83, 1.32	.706
		SIMD (*range* = quintile 1–5)	0.96	0.84, 1.14	7.44
		Current/past mental health support (*n* = 121)	1.44	0.90, 2.33	.132
		Additional support for learning in school (*n* = 85)	1.76	1.03, 3.00	**.038**
		Keyworkers (*n* = 206)	1.44	0.96, 2.15	.079
		Poor relationships at home since Covid-19 (*n* = 35)	1.92	0.91, 4.05	.089
		Much reduced physical activity since Covid-19 (*n* = 107)	1.22	0.75, 1.97	.420

*n* = number of participants.

#### Depression

The first step of the regression (age, gender female and SIMD) was significant, omnibus χ^2^ = (3, N = 542) = 32.38, *p* = < .001, and accounted for 13% of the variance (Nagelkerke’s R^2^) in depression scores. As shown in Table 1, risk of depression increased with age (adjusted OR = 2.55, *p* < .001), with a one-unit increase in age leading to a 2.55 fold increase in the odds of depression. The final model of the regression (age, gender female, SIMD, metal health support, additional support in school, poor relationships at home since Covid-19 and much reduced physical activity since Covid-19) was significant, omnibus χ^2^ = (8, N = 542) = 92.15, *p* = < .001, accounting for 34% of the variance (Nagelkerke’s R2) in depression scores (an increase of 21%). As shown in Table 1, age remained significant (adjusted OR = 2.97, *p* < .001) and risk of depression was found to be higher for young people who currently or previously received mental health support (adjusted OR = 3.76, *p* < .001), young people who were receiving additional support in school prior to Covid-19 (adjusted OR = 3.22, *p* = .002) and young people who reported poorer relationships at home since Covid-19 (adjusted OR = 8.59, *p* < .001). There was no association between female gender, SIMD, keyworker status or reduced physical activity since the Covid-19 pandemic and depression.

#### Anxiety

The first step of the regression (age, gender female and SIMD) was significant, omnibus χ^2^ = (3, N = 542) = 18.06, *p* = < .001, and accounted for 9% of the variance (Nagelkerke’s R^2^) in anxiety scores. As shown in Table 1, risk of depression increased with age (adjusted OR = 1.74, *p* = .002), with a one-unit increase in age leads to a 1.74 fold increase in the odds of anxiety. The final model of the regression (age, gender female, SIMD, metal health support, additional support in school, poor relationships at home since Covid-19 and much reduced physical activity since Covid-19) was significant, omnibus χ^2^ = (8, N = 542) = 39.58, *p* = .001, accounting for 19% of the variance (Nagelkerke’s R2) in anxiety scores (an increase of 10%). As shown in Table 1, age remained significant (adjusted OR = 2.03, *p* = .002) and risk of anxiety was found to be higher for young people who currently or previously received mental health support (adjusted OR = 3.50, *p* = .002) and young people who reported poorer relationships at home since Covid-19 (adjusted OR = 3.75, *p* = .029). There was no association between female gender, SIMD, receiving additional support in school prior to Covid-19, keyworker status, or reduced physical activity since the Covid-19 pandemic and depression.

#### PTSD-like symptoms about Covid-19

The first model of the regression (age, gender female and SIMD) was significant, omnibus χ^2^ = (3, N = 542) = 20.00, *p* = < .001, accounting for 5% of the variance (Nagelkerke’s R^2^) in PTSD-like symptoms about Covid-19 scores. As shown in Table 1, risk of PTSD-like symptoms about Covid-19 was found to be greater in females (adjusted OR = 2.63, *p* < .001). The final model of the regression (age, gender female, SIMD, mental health support, additional support in school, poor relationships at home since Covid-19 and much reduced physical activity since Covid-19) was significant, omnibus χ^2^ = (8, N = 542) = 38.24, *p* = .003, accounting for 10% of the variance (Nagelkerke’s R2) in anxiety scores (an increase of 5%). As shown in Table 1, female gender remained significant (adjusted OR = 2.49, *p* < .001) and risk of avoidance and intrusion was found to be higher for young people who were receiving additional support in school prior to Covid-19 (adjusted OR = 1.76, *p* = .038). There was no association between age, SIMD, current or past mental health support, keyworker status, poor home relationships or reduced physical activity since the Covid-19 pandemic and PTSD-like symptoms about Covid-19.

### Perceived changes in mental health

**Fig 1:**
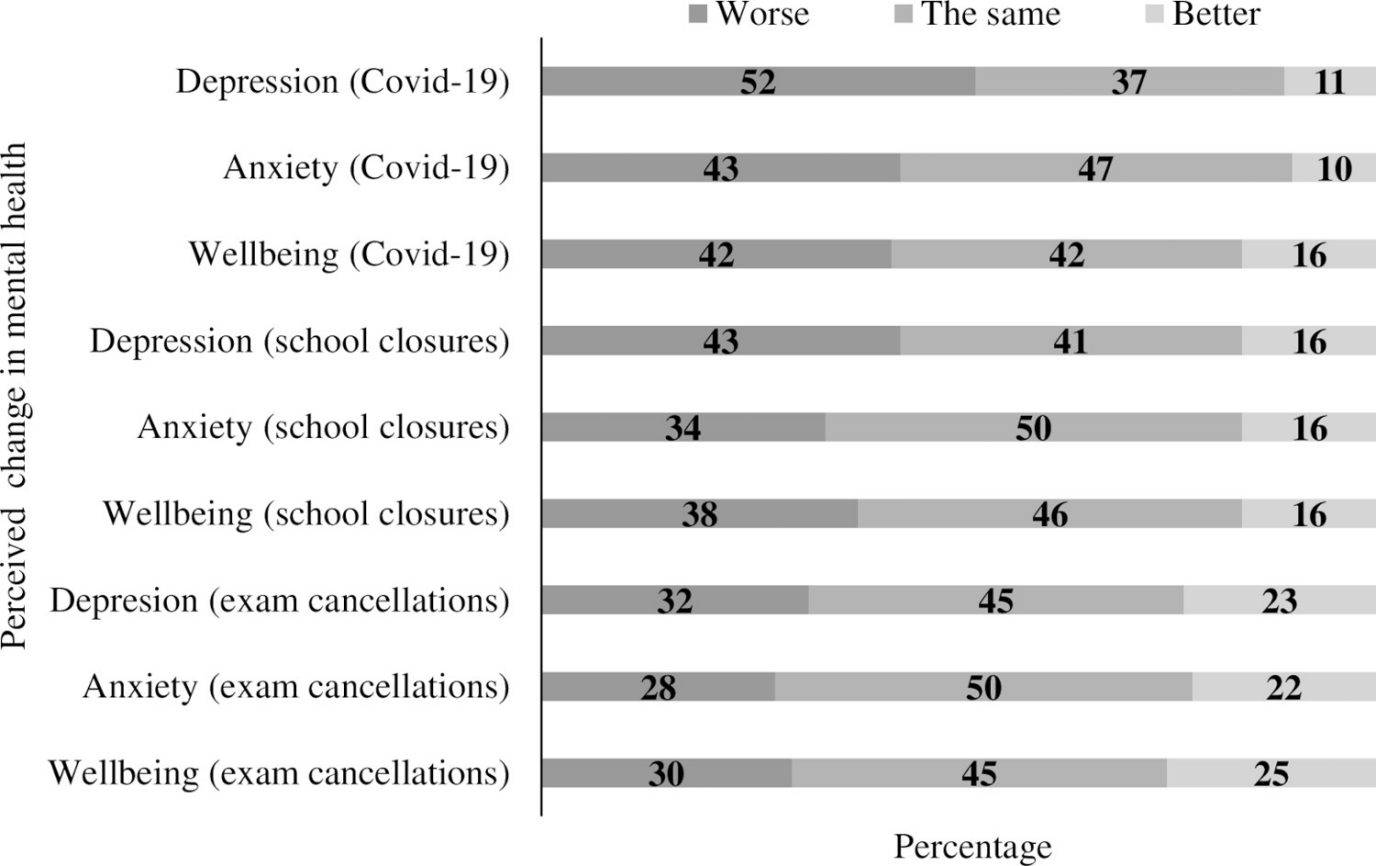
Perceived changes in depression, anxiety and wellbeing due to Covid-19, school closures and exam cancellations.

Perceived changes in mental health and wellbeing due to Covid-19, school closures and exam cancellations can be found in Fig 1. Perceived changes (*n* and %) split by gender and clinical status can be found in supporting information (S1 Table), with statistically significant differences between males-females and within clinical status reported below.

#### Perceived change in depression, anxiety and wellbeing since the Covid-19 outbreak, due to school closures and exam cancellations by gender

Perceptions of change in depression since Covid-19 differed by gender, χ^2^ (2, N = 729) = 31.98, *p* < .001, *V* = .209, with females more likely (59%) than males (39%) to perceive symptoms of depression to have got worse and males more likely (50%) than females (30%) to say that depression levels remained the same. Perceptions of change in anxiety since Covid-19 also differed by gender, χ^2^ (2, N = 729) = 14.49 *p* = .001, *V* = .141, with males more likely (56%) to perceive symptoms of anxiety to remain the same and less likely (34%) to perceive symptoms of anxiety to have got worse since the Covid-19 outbreak. Perceptions of change in anxiety due to school closures further differed by gender, χ^2^ (2, N = 729) = 14.35, *p* = .001, *V* = .140, with males more likely (59%) to perceive symptoms of anxiety to remain the same and less likely (26%) to perceive symptoms of anxiety to have got worse due to school closures.

#### Perceived change in depression, anxiety and wellbeing since the Covid-19 outbreak, due to school closures and exam cancellations by clinical status

Adolescents who met the clinical depression threshold were more likely (80%) to state that symptoms of depression got worse and less likely (14%) to say they remained the same since the Covid-19 outbreak, χ^2^ (2, N = 807) = 23.50, *p* < .001, *V* = 171. They were also more likely (59%) to state that symptoms of depression got worse and less likely (28%) to say they remained the same due to school closures, χ^2^ (2, N = 807) = 7.95, *p* = .019, *V* = .099, and were more likely (49%) to state that their symptoms of depression got worse and less likely (32%) to say they remained the same due to exam cancellations, χ^2^ (2, N = 807) = 10.84, *p* < .004, *V* = .116.

Similarly, adolescents who met clinical anxiety threshold were more likely (63%) to state that their symptoms of anxiety got worse and less likely (27%) to say they remained the same since the Covid-19 outbreak, χ^2^ (2, N = 805) = 10.11, *p* = .006, *V* = .112. They were also more likely (50%) to state that symptoms of anxiety got worse and less likely to say they remained the same (23%) or got better (27%) due to school closures, χ^2^ (2, N = 805) = 16.83, *p* < .001, *V* = 135. Furthermore, adolescents who met the clinical anxiety threshold were more likely (43%) than expected to state that their symptoms of anxiety got worse due to exam cancellations, χ^2^ (2, N = 805) = 7.74, *p* < .021, *V* = .098.

Adolescents who met either (or both) clinical depression or anxiety threshold were more likely (66%) to state that their wellbeing got worse and less likely (26%) to say they remained the same or got better (9%) since the Covid-19 outbreak, χ^2^ (2, N = 784) = 24.58, *p* < .001, *V* = .117, and were more likely (52%) to state that their wellbeing got worse and less likely to say they remained the same (32%) due to school closures, χ^2^ (2, N = 784) = 9.42, *p* = .009, *V* = .110. Furthermore, adolescents who met either (or both) clinical depression or anxiety threshold were more likely (42%) than expected to state that their wellbeing got worse due to exam cancellations, χ^2^ (2, N = 784) = 7.24, *p* < .027, *V* = .096.

## Discussion

We investigated rates of depression, anxiety and PTSD-like symptoms avoidance and intrusion specifically about Covid-19. We explored whether current or past mental health support, additional support in school, keyworker status, poorer household relationships since the outbreak of Covid-19 or reduced physical activity since the outbreak of Covid-19 was associated with elevated levels of depression, anxiety or PTSD-like symptoms in adolescents. Lastly, we explored adolescents’ perceptions of changes in their mental health and wellbeing due to the Covid-19 pandemic, school closures and the cancellation of exams. We discuss the findings below.

### Mental health rates

The results showed that 9% (13% if including borderline cases) of our sample met clinical threshold levels for depression and 7% for anxiety (13% if including borderline cases). While rates of depression and anxiety have been found to differ across pre-pandemic studies, epidemiological studies have suggested depression rates of 2.1% and anxiety rates of 7.2% [9], suggesting depression rates in the current sample are greater than pre-pandemic levels while anxiety rates remained stable. This is a similar trend to that found in a recent study with UK children that found increases in depression but not anxiety pre-and-during Covid-19 [19]. Rates for both depression and anxiety in the current study were however lower in comparison to adolescent data reported in England (14% depression and 10% anxiety) [8]. Rates of elevated avoidance and intrusion were also high in the current sample, with 29% reporting elevated PTSD-like symptoms about Covid-19. This is similar to rates reported during Covid-19 in Malaysia (25.4%) [10] and Italy (30.9%) [11]. While the current findings provide a snapshot in time, of clinically relevant rates, caution must be given to the interpretation of the findings. It is possible that the Covid-19 pandemic and mitigations that followed have exacerbated already increasing trends in mental ill-health in adolescents. Future research, particularly epidemiological studies, with representative samples, are now warranted to determine changes in the mental health of adolescents. Similarly, investigation of trajectories across age and gender will allow for targeted intervention.

### Predictors of depression, anxiety, and avoidance and intrusion

We also investigated the association between potential Covid-19 related risk factors on adolescent depression, anxiety and PTSD-like symptoms about Covid-19. Similar to de Miranda et al. [37], we found that older adolescents were two to three times more likely to meet clinical thresholds for depression and anxiety. However, unlike previous literature [8, 38], we did not find females to be more at risk of depression or anxiety, although females were almost three times more likely to report elevated avoidance and intrusive thoughts about Covid-19. This is a comparable finding to pre-pandemic data that has shown female adolescents were more likely to show post-traumatic stress symptoms in adolescence [39].

Our finding that adolescents who were currently or previously receiving mental health support had almost four times the odds of reaching clinical cut-off threshold for depression and anxiety, echoes Mansfield et al.’s [8] finding that adolescents in England who had accessed mental health support had almost four times the odds of meeting clinical cut-off thresholds for depression and anxiety. These findings add to the growing body of literature that suggests adolescents with pre-existing symptoms have been uniquely, and negatively, impacted by the Covid-19 pandemic [23, 40]. Furthermore, adolescents who were receiving additional support in school prior to Covid-19 had almost four times the odds of meeting clinical cut-off thresholds for depression and almost two times the odds of reporting elevated avoidance and intrusive thoughts about Covid-19. Our finding extends previous research with children with special educational needs and disabilities [22] and children and young people with pre-existing mental health problems [23] and highlights the negative impact on adolescent mental health during the Covid-19 outbreak and extends to adolescents receiving broader additional support in school.

We further highlighted that young people who reported poorer relationships at home since Covid-19 had almost nine times the odds of reaching clinical cut-off thresholds for depression and almost four times the odds of reaching clinical cut-off thresholds for anxiety. Recent research has reported more strained family relationships, higher parenting irritability, couple conflict and lower family positive expressiveness since the Covid-19 pandemic [41] and we know from pre-pandemic research that child adjustment is, in part, dependent on relationships in a family [42, 43]. Furthermore, while there is variability in how families have been impacted by the Covid-19 pandemic, the social and economic impact of Covid-19 has been severe and widespread, and previous research has shown that family adversity can have negative consequences for children and young people [44]. Indeed, previous research has shown that economic pressure is related to family distress and parenting practices [45] and it may be that our finding is related to these difficulties. However, future research that investigates *why* poor relationships during Covid-19 have negatively impacted young people’s mental health are now warranted in order to develop Covid-19 related interventions to support families.

We did not find an association between changes in physical activity since the Covid-19 outbreak or keyworker status on any mental health outcome. While the relationship between physical activity and mental health is less well documented in the child and adolescent literature than it is in adult populations, physical activity has been shown to reduce depression and anxiety [46, 47]. The lack of association in the current study may be explained in a number of ways. Adolescents were asked to self-report changes in physical activity, which may have resulted in over-or-under estimations of energy expenditure of inactivity [48]. It is also possible that the reduction in physical activity since the Covid-19 pandemic was not severe enough, or present for long enough to exert an effect on mental health outcomes. Given reports of reduced physical activity during the Covid-19 pandemic [49], further research, particularly longitudinal research examining physical activity and mental health outcomes in adolescence is warranted.

The explanation of a lack of association between keyworker status and mental health outcomes appear to represent the mixed research literature. For example, while research with young people whose parents were keyworkers has found greater depression and anxiety symptoms [8, 50], other research has found increases in depression and post-traumatic stress symptoms of which no increases were moderated by worker status of a parent [18]. It is possible that the association between parent keyworker status and mental health outcomes reported in previous studies does not extend to adolescents who are keyworkers, however, this area of research is in its infancy and further research is warranted.

### Perceived changes in mental health

Adolescents, in the current study, were also asked whether they perceived the Covid-19 pandemic, school closures or exam cancelations to have impacted their mental health. The findings show that 52% of adolescents reported worsening of feelings of depression, 43% worsening of feelings of anxiety and 42% reported poorer wellbeing since the Covid-19 pandemic. This finding adds to earlier research by the Scottish Youth Parliament, YouthLink Scotland and Young Scot [30] who reported that almost two-fifths (39%) of adolescents said they were moderately or extremely concerned about their mental wellbeing during the Covid-19 pandemic. The finding that females were more likely than males to report worsening of depression likely reflects the dominance of depression in females [51]. Adolescents who were experiencing clinical levels of depression and anxiety were particularly likely to report worsening of their mental health, specifically, 80% reported worsening of symptoms of depression, 63% reported worsening of anxiety and 66% reported poorer wellbeing since the Covid-19 pandemic. Adolescents’ own perceptions of their mental health mirrors research suggesting that young people who are experiencing clinical levels of symptoms are more severely impacted by the Covid-19 outbreak and the mitigations that followed [23].

Our findings also show that almost half (43%) of adolescents reported worsening of feelings of depression, over one-third (34%) reported worsening of feelings of anxiety and over one-third (38%) reported poorer wellbeing due to school closures. Adolescents who were experiencing clinical levels of symptoms were particularly impacted by school closures, with 59% reporting worsening of symptoms of depression, 50% reporting worsening of anxiety and 52% reporting poorer wellbeing due to school closures. For some children and adolescents, education offers a safe environment, and school closures due to Covid-19 have been shown to negatively impact mental as well as physical health [52]. Our findings add to the growing literature base of the negative impact of school closures and support calls to ensure that school closures are only enacted for the benefit of children and young people and as a last resort [53]. It is however important to note that for some adolescents (16%), the closure of schools had a positive impact on their mental health. We encourage educators and policy makers to consider this in discussions about recovery from Covid-19, and how this might inform, for example, support structures in school.

Adolescents, in the current study, appeared to have mixed feelings about the cancellation of exams, with some young people stating that exam cancellations had a negative impact on their mental health while for others this was a positive experience (i.e. depression: 32% worse vs. 22% better; anxiety: 27% worse vs. 21% better; wellbeing: 30% worse vs. 25% better). These findings may relate to separate research that also reported mixed findings, with 21% of young people in England stating they were happy that exams were cancelled and 46% stating they would have preferred to sit their exams [54]. The negative impact of exam cancellations however was particularly noted by adolescents who were experiencing clinically significant symptoms. Specifically, 49% reported worsening of depression, 43% worsening of anxiety and 42% reported poorer wellbeing due to the cancellation of exams. Previous research has highlighted early concerns by young people about the cancellation of exams [30] with others noting that teachers may find it difficult to grade and rank students accurately [55]. It is important to note, that in the current study, young people responded to questions about exam cancellations *after* the release of their grades and therefore the current findings are not compounded by levels of uncertainty surrounding how exams would be graded. Given the mixed findings of the impact of the cancellations of exams, we would encourage scholars and policy makers to investigate this further, to inform any new provision around assessment and the use of examinations.

### Limitations and conclusion

This study provides a novel contribution to the field, highlighting rates of depression, anxiety and elevated PTSD-like symptoms avoidance and intrusive thoughts about Covid-19 in adolescents in Scotland, demonstrating perceived changes in mental health due to Covid-19, school closures and exam cancellations, and the specific impact of these on young people with clinical threshold levels of depression and anxiety. The findings also highlight the range of contextual and Covid-19 specific factors associated with increased odds of clinically relevant depression, anxiety and PTSD-like symptoms avoidance and intrusive thoughts about Covid-19. The study does however have a number of limitations. While commissioned as a rapid review project, providing vital data on the mental health and education of adolescents during the Covid-19 pandemic, the findings cannot infer causality. It is also limited by convenience sampling and some single-item questions. Single-item questions can have lower sensitivity (i.e. due to fewer points of discrimination), produce low content validity and lack a measure of internal consistency, which may bias the data. However, given the lack of standardised measures available and the importance of these questions (i.e. in relation to Covid-19), it was deemed necessary to include these but offer caution to confirm the findings. Future work, with a focus on mixed and multiple methodologies, with longitudinal designs, supporting high quality scientifically rigorous research is now needed to understand the mechanisms in which these factors lead to difficulties with mental health, and to investigate what factors can mitigate these risks. In doing so, this will inform national policy in supporting young people’s mental health and recovery from the Covid-19 pandemic.

## Supporting information

S1 Table Percentages of perceived changes in mental health, by gender and clinical status. (DOCX)
