# Supplementary material for: Rates, perceptions and predictors of depression, anxiety and Post Traumatic Stress Disorder (PTSD)-like symptoms about Covid-19 in adolescents
Source: PLoS One. 2022 Apr 27;17(4):e0266818. doi: 10.1371/journal.pone.0266818 (PMC9045622; doi:10.1371/journal.pone.0266818)
Supplement: S1 Table — (DOCX) [file pone.0266818.s001.docx]

S1 Table. Percentages of perceived changes in mental health, by gender and clinical status

|  | **Gender** | | | | **Clinical threshold** | | |
| --- | --- | --- | --- | --- | --- | --- | --- |
| **Perceptions of change since Covid-19:** | **Female**  **% (*n*)** | **Male**  **% (*n*)** | **Total**  **% (*n*)** | **Yes**  **% (*n*)** | | **No**  **% (*n*)** | **Total**  **% (*n*)** |
| Depression (worse) | 59 (*n* = 274)* | 39 (*n* =103)** | *52 (n* = 377) | 80 (*n* = 55)** | | 49 (*n* =364) | 52 (*n* = 419) |
| Depression (same) | 30 (*n* = 140)** | 50 (*n* = 134)** | 37 (*n* = 274) | 14 (*n* = 10)** | | 40 (*n* = 292) | 37 (*n* = 302) |
| Depression (better) | 11 (*n* = 48) | 11 (*n* = 30) | 11 (*n* = 78) | 6 (*n* = 4) | | 11 (*n* = 82) | 11 (*n* = 86) |
| Depression total | 63 (*n* = 462) | 37 (*n* = 267) | 100 (*n* = 729) | 9 (*n* = 69) | | 91 (*n* = 738) | 100 (*n* = 807) |
| Anxiety (worse) | 48 (*n* = 222) | 34 (*n* =90)* | 43 (*n* = 312) | 63 (*n* = 35)* | | 42 (*n* =316) | 43 (*n* = 351) |
| Anxiety (same) | 43 (*n* = 198) | 56 (*n* = 149)* | 47 (*n* = 347) | 27 (*n* = 15)* | | 48 (*n* = 361) | 47 (*n* = 376) |
| Anxiety (better) | 9 (*n* = 42) | 10 (*n* = 28) | 10 (*n* = 70) | 11 (*n* = 6) | | 10 (*n* = 72) | 10 (*n* = 78) |
| Anxiety total | 63 (*n* = 462) | 37 (*n* = 267) | 100 (*n* = 729) | 7 (*n* = 56) | | 93 (*n* = 749) | 100 (*n* = 805) |
| Wellbeing (worse) | 45 (*n* = 210) | 35 (*n* =93) | 42 (*n* = 303) | 66 (*n* = 61)*** | | 39 (*n* = 267) | 42 (*n* =328) |
| Wellbeing (same) | 38 (*n* = 174) | 49 (*n* = 131) | 42 (*n* = 305) | 26 (*n* =24)* | | 44 (*n* = 303) | 42 (*n* = 327) |
| Wellbeing (better) | 17 (*n* = 78) | 16 (*n* = 43) | 16 (*n* = 121) | 9 (*n* = 8)* | | 17 (*n* = 121) | 16 (*n* = 129) |
| Wellbeing total | 63 (*n* = 462) | 37 (*n* = 267) | 100 (*n* = 729) | 12 (*n* = 93) | | 88 (*n* = 691) | 100 (*n* = 784) |
| **Perceptions of change due to school closures:** | **Female**  **% (*n*)** | **Male**  **% (*n*)** | **Total**  **% (*n*)** | **Yes**  **% (*n*)** | | **No**  **% (*n*)** | **Total**  **% (*n*)** |
| Depression (worse) | 47 (*n* = 215) | 38 (*n* = 102) | 43 (*n* = 317) | 59 (*n* = 738)* | | 42 (*n* =310) | 43 (*n* = 351) |
| Depression (same) | 37 (*n* = 169) | 48 (*n* = 129) | 41 (*n* = 298) | 28 (*n* = 19)* | | 42 (*n* = 308) | 41 (*n* = 327) |
| Depression (better) | 17 (*n* = 78) | 13 (*n* = 36) | 16 (*n* = 114) | 13 (*n* = 9) | | 16 (*n* =120) | 16 (*n* =129) |
| Depression total | 63 (*n* = 462) | 37 (*n* = 267) | 100 (*n* = 729) | 9 (*n* = 69) | | 91 (*n* = 738) | 100 (*n* = 807) |
| Anxiety (worse) | 38 (*n* = 175) | 26 (*n* = 69)* | 33 (*n* =244) | 50 (*n* = 28)* | | 33 (*n* = 244) | 34 (*n* = 272) |
| Anxiety (same) | 45 (*n* = 207) | 59 (*n* = 157)* | 50 (*n* = 364) | 23 (*n* = 13)** | | 52 (*n* = 386) | 50 (*n* = 399) |
| Anxiety (better) | 17 (*n* = 80) | 15 (*n* = 41) | 17 (*n* = 121) | 27 (*n* = 15)* | | 16 (*n* = 119) | 16 (*n* = 134) |
| Anxiety total | 63 (*n* = 462) | 37 (*n* = 267) | 100 (*n* = 729) | 7 (*n* = 56) | | 93 (*n* = 749) | 100 (*n* = 805) |
| Wellbeing (worse) | 41 (*n* = 190) | 33 (*n* = 87) | 38 (*n* = 227) | 52 (*n* = 48)* | | 36 (*n* = 250) | 38 (*n* = 298) |
| Wellbeing (same) | 42 (*n* = 196) | 51 (*n* = 137) | 46 (*n* = 33) | 32 (*n* = 30)* | | 48 (*n* = 330) | 46 (*n* = 360) |
| Wellbeing (better) | 16 (*n* = 76) | 16 (*n* = 43) | 16 (*n* = 119) | 16 (*n* = 15) | | 16 (*n* = 111) | 16 (*n* = 126) |
| Wellbeing total | 63 (*n* = 462) | 37 (*n* = 267) | 100 (*n* = 729) | 12 (*n* = 93) | | 88 (*n* = 691) | 100 (*n* = 784) |
| **Perceptions of change due to exam cancelations:** | **Female**  **% (*n*)** | **Male**  **% (*n*)** | **Total**  **% (*n*)** | **Yes**  **% (*n*)** | | **No**  **% (*n*)** | **Total**  **% (*n*)** |
| Depression (worse) | 36 (*n* = 165) | 27 (*n* = 72) | 32 (*n* = 237) | 49 (*n* = 34)** | | 30 (*n* = 222) | 32 (*n* = 256) |
| Depression (same) | 43 (*n* = 197) | 51 (*n* = 135) | 46 (*n* = 332) | 32 (*n* = 22)* | | 46 (*n* = 340) | 45 (*n* = 362) |
| Depression (better) | 22 (*n* = 100) | 22 (*n* = 60) | 22 (*n* = 160) | 19 (*n* = 13) | | 24 (*n* = 176) | 23 (*n* = 189) |
| Depression total | 63 (*n* = 462) | 37 (*n* = 267) | 100 (*n* = 729) | 9 (*n* = 69) | | 91 (*n* = 738) | 100 (*n* = 807) |
| Anxiety (worse) | 29 (*n*= 135) | 24 (*n* = 64) | 27 (*n* = 199) | 43 (*n* = 24)* | | 27 (*n* = 204) | 28 (*n* = 28) |
| Anxiety (same) | 50 (*n* = 231) | 55 (*n* = 147) | 52 (*n* = 378) | 34 (*n* = 19) | | 51 (*n* = 385) | 50 (*n* = 44) |
| Anxiety (better) | 21 (*n* = 96) | 21 (*n* = 46) | 21 (*n* = 152) | 23 (*n* = 13) | | 21 (*n* = 160) | 22 (*n* = 173) |
| Anxiety total | 63 (*n* = 462) | 37 (*n* = 267) | 100 (n = 729) | 7 (*n* = 56) | | 93 (*n* = 749) | 100 (*n* = 805) |
| Wellbeing (worse) | 34 (*n* = 156) | 24 (*n* = 64) | 30 (*n* + 220) | 42 (*n* = 39)* | | 28 (*n* = 196) | 30 (*n* = 235) |
| Wellbeing (same) | 43 (*n* = 197) | 50 (*n* = 133) | 45 (*n* = 330) | 37 (*n* = 34) | | 46 (*n* = 320) | 45 (*n* = 354) |
| Wellbeing (better) | 24 (*n* = 109) | 26 (*n* = 70) | 25 (*n* = 179) | 22 (*n* = 20) | | 25 (*n* = 175) | 25 (*n* = 195) |
| Wellbeing total | 63 (*n* = 462) | 37 (*n* = 267) | 100 (*n* = 729) | 12 (*n* = 93) | | 88 (*n* = 691) | 100 (*n* = 784) |

**p*<0.5, ** *p*<.01, *** *p*<.001

*n* = number
